# Comparative Studies on Two-Electrode Symmetric Supercapacitors Based on Polypyrrole:Poly(4-styrenesulfonate) with Different Molecular Weights of Poly(4-styrenesulfonate)

**DOI:** 10.3390/polym11020232

**Published:** 2019-02-01

**Authors:** Hoseong Han, Jun Seop Lee, Sunghun Cho

**Affiliations:** 1 School of Chemical Engineering, Yeungnam University, Gyeongsan 38541, Korea; byecome123@gmail.com; 2 Department of Materials Science and Engineering, College of Engineering, Gachon University, Seongnam 13120, Korea; junseop@gachon.ac.kr

**Keywords:** supercapacitor, coin cell, conducting polymer, polypyrrole, pss

## Abstract

Poly(4-styrenesulfonate)-conducting polymer (PSS-CP) is advantageous for thin-film electrode manufacturing due to its high conductivity, high charge storage, structural stability, and excellent ink dispersion. In this work, comparative studies of two-electrode symmetric supercapacitors using Polypyrrole:Poly(4-styrenesulfonate) (PPy:PSS), with different molecular weights (*M*_w_’s) of Poly(4-styrenesulfonate) (PSS) as the electrodes, were performed. PPy:PSS can be easily prepared using a simple solution process that enables the mass production of thin-film electrodes with improved electrical and electrochemical properties. As-prepared PPy:PSS, with different PSS molecular weights, were assembled into two-electrode supercapacitors based on coin cell structures. It was confirmed that the electrical and electrochemical properties of PPy:PSS were improved with increasing PSS molecular weight. The coin cell, using PPy:PSS with a PSS molecular weight of 1.0 × 10^6^ g/mol, exhibited higher areal capacitance (175.3 mF/cm^2^), higher volumetric capacitance (584.2 F/cm^3^), and longer cycling stability (86.3% after 5000 cycles) compared to those of PPy:PSS with PSS molecular weights of 2.0 × 10^5^ and 7.0 × 10^4^ g/mol. This work provides an efficient approach for producing cost-effective and miniaturized supercapacitors with high conductivity and high specific capacitance for practical applications in a variety of electronic devices.

## 1. Introduction

Conducting polymers (CPs) are defined as polymers that can conduct electricity and have the inherent properties of polymers, such as processability, flexibility, and a low unit cost [1]. Due to these advantages of CPs, p-type CPs, such as polypyrrole (PPy), polyaniline (PANI), polythiophene (PT), and poly(3,4-ethylenedioxythiophene) (PEDOT), have attracted much interest as fascinating electrode materials in practical applications, including supercapacitors [2,3,4,5,6,7], sensors [8,9,10,11], solar cells [12,13,14,15], thin-film transistors [16,17,18], and smart windows [7,19,20]. Among the various CPs, PPy that consists of five-membered heterocyclic rings is highly attractive as an electrode material for supercapacitor and battery applications because of its facile synthesis, superior flexibility, and environmental stability compared to those of most CPs [1,3,4,5,6,21]. Hence, various efforts have been made to produce PPy nanomaterials, including nanoparticles (NPs), nanotubes (NTs), nanorods (NRs), and core-shells, through chemical oxidation polymerization and electrochemical deposition [1,21,22]. The widely-used method of Jang et al. has been used to fabricate PPy NPs, NTs, and NRs with precisely controlled morphologies using microemulsion polymerization [1,21]. These fascinating features of PPy nanomaterials have stimulated the growing interest in high-performance supercapacitors using PPy nanomaterials as electrodes [2,3,4,5,6,7].

Supercapacitors are attractive and promising energy storage devices due to their faster charging process, higher power density, superior low-temperature performance, and longer life cycles compared to those of Li-ion batteries [2,3,23,24,25,26,27]. In particular, pseudocapacitors including CPs and metal oxides offer higher specific capacitance and larger energy density than the electric double layer capacitors (EDLCs), such as graphene, carbon nanotubes (CNTs), and activated carbon (AC) [2,3,23,24,25,26,27]. Therefore, many studies have been performed on supercapacitors using PPy materials as the electrodes [2,3,4,5,6,7]. The sandwiched PPy film/MoS_2_ monolayer nanocomposites were used as the electrodes in a symmetric supercapacitor [4]. Huang et al. reported a NiCo_2_S_4_@PPy core-shell electrode with a specific capacitance of 9.781 F/cm^2^ at 5 mA/cm^2^ and a capacitance degradation of 19.36% after 2500 cycles [5]. However, the reversible doping/de-doping process of ions usually changes the physical structure of PPy and, therefore, conducting polymers including PPy are often degraded after less than 1000 supercapacitor cycles [2,3,4,5,6,7]. Despite these achievements, the stable dispersion, easy film formation, and structural stability of PPy nanomaterials have become recognized as important issues for the manufacturing of high-performance supercapacitors.

Poly(4-styrenesulfonate) (PSS), a water-dispersible anionic polymer, has attracted intense attention because it enables simple film formation and the improved dispersion of CP nanomaterials [11,12,15,19,28,29]. In addition, PSS promotes the formation of head-to-tail structures in CP chains, as well as the improved dispersion of CPs in water [11,12,15,19,28,29]. Thus, PSS has been regarded as an effective additive for preparing water-dispersible CP solutions for their use as electrode materials. Poly(3,4-ethylene dioxythiophene):Poly(4-styrenesulfonate) (PEDOT:PSS) has been the most widely commercialized CP because of its high processability, small bandgap (1.6–1.7 eV), and good optical properties [12,15,19,28]. It is known that the conductivity of PEDOT:PSS can be enhanced to 10^3^ S/cm by treatment with 5% dimethyl sulfoxide (DMSO) [12,15,19,28]. Despite its excellent conductivity, PEDOT:PSS exhibits an inferior redox reaction compared to PPy and polyaniline (PANI), resulting in a poor specific capacitance and low reliability during repeated cycling [2,3,4,5,6,7]. To overcome the limitations of PEDOT:PSS, Polyaniline:Poly(4-styrenesulfonate) (PANI:PSS) has been suggested as an efficient alternative to PEDOT:PSS [11,29]. PANI:PSS provides an excellent redox reaction and better charge storage characteristics compared to PEDOT:PSS [11,29]. However, PANI-based supercapacitors usually suffer from chain scissions and volume changes during the repeated adsorption/desorption of electrolyte ions [2,3,30]. Therefore, it is necessary to develop a PSS-based CP that can overcome the limitations of PANI:PSS and PEDOT:PSS. Although Polypyrrole:Poly(4-styrenesulfonate) (PPy:PSS) has been applied as a cathode material in lithium-sulfur batteries and as a biocompatible electrode, no studies have been reported on the effect of the molecular weight (*M*_w_) of PSS on the electrical and electrochemical performances of PPy:PSS [31,32,33]. Therefore, it is expected that the limitations of conventional pseudocapacitors, based on either PEDOT:PSS or PANI:PSS, can be overcome by combining the advantages of PPy:PSS and a two-electrode supercapacitor.

Here, we report on the preparation of PPy:PSS inks, with different molecular weights of PSS, for their use as electrode materials in a two-electrode symmetric supercapacitor. Since the coin cell is more practical for evaluating actual capacitive performance than a three-electrode supercapacitor, the PPy:PSS electrode was assembled into a coin cell [26]. The particle size of PPy:PSS increases with increasing PSS molecular weight, resulting in a higher protonation level and improved PPy:PSS conductivity. This work focuses on identifying the optimal PSS molecular weight to enhance the electrical and electrochemical performances of supercapacitors made from PPy:PSS electrodes. PSS molecular weights of 7.0 × 10^4^, 2.0 × 10^5^, and 1.0 × 10^6^ g/mol were used to polymerize PPy. A variety of analytical methods, including field emission scanning electron microscopy (FE-SEM), the four-point probe method, X-ray photoelectron spectroscopy (XPS), cyclic voltammetry (CV), electrochemical impedance spectroscopy (EIS), and galvanostatic charge/discharge (GCD) analyses, were used to observe the dependence of the electrical and electrochemical performance characteristics of PPy:PSS on its molecular weight. The PPy:PSS with the PSS molecular weight of 1.0 × 10^6^ g/mol (PPy100) was found to be advantageous for the construction of coin cell supercapacitors, demonstrating superior electrical and electrochemical performance characteristics compared to the PSS molecular weights of 2.0 × 10^5^ g/mol (PPy20) and 7.0 × 10^4^ g/mol (PPy7).

## 2. Materials and Methods

### 2.1. Materials

Pyrrole (99%), iron (iii) chloride hexahydrate (FeCl_3_·6H_2_O, 97%), and PSS solution with different molecular weights (7.0 × 10^4^, 2.0 × 10^5^, and 1.0 × 10^6^ g/mol) were acquired from Sigma-Aldrich (St. Louis, MO, USA). Potassium hydroxide (KOH) was purchased from Daejung Chemical & Metals Co., Ltd. (Siheung, Korea). The components for coin cell assembly (CR2032 type) and nickel (Ni) foil (thickness: 0.03 mm) were obtained from the MTI Corporation (Richmond, CA, USA). The polypropylene-polyethylene-polypropylene (PP-PE-PP) trilayer (thickness: 25 μm) was obtained from Celgard, LLC. (Charlotte, NC, USA).

### 2.2. Fabrication of PPy:PSS and Characterizations of PPy:PSS

Pyrrole monomer (0.30 g) was added to 80 mL of FeCl_3_·6H_2_O solution (0.7 wt % with respect to distilled water), followed by vigorous stirring at 3 °C for 3 hours. After the polymerization of pyrrole, the mixture of PPy was centrifuged to obtain dark precipitates of PPy. Next, 0.5 g of PPy precipitates were dissolved in 40 mL of PSS solution (1.4 wt % with respect to distilled water) and stirred vigorously at room temperature for 3 hours. As-prepared PPy:PSS solution was sonochemically treated using an ultrasonic bath (CPX2800H-E, Branson Ultrasonics Co., Danbury, CT, USA) with 110 W of power and a frequency of 40 kHz. Morphological images and diameter distributions of PPy:PSS were recorded using an FE-SEM instrument (S-4800, HITACHI, LTD, Hitachi, Japan). An investigation into the chemical compositions and protonation states of the PPy:PSS was conducted using a K-Alpha XPS instrument (Thermo K-Alpha XPS, Thermo Fisher Scientific, Waltham, MA, USA.). The electrical conductivity of the PPy:PSS was measured using a four-point probe station (Mode Systems Co., Hanam, Korea) equipped with a Keithley 2400 current source meter (Keithley Co., Cleveland, OH, USA) [34]. To identify the redox behaviors of pure PPy:PSS, cyclic voltammograms (CVs) of PPy:PSS were evaluated using a ZIVE SP2 electrochemical workstation (Wonatech, Seoul, Korea). The CVs of pure PPy:PSS electrodes were measured from 0 to 1.0 V at a rate of 20 mV/s in 1 M KOH electrolyte. The reference electrode and the counter electrode were Ag/AgCl in saturated KCl solution and a platinum sheet (2.0 × 2.0 cm^2^), respectively. A PPy:PSS film with a thickness of approximately 3 μm was coated onto a glass substrate and an as-prepared PPy:PSS-coated electrode was used as the working electrode for the CV measurements.

### 2.3. Assembly of Coin Cells Containing PPy:PSS

Firstly, 0.2 mL of PPy:PSS solution was spread onto Ni-foil substrates by a spin-coater (Spin-1200D, Midas Systems Co., Ltd., Daejeon, Korea) and the PPy:PSS samples were dried at 25 °C for 20 min using a vacuum oven to evaporate the water. The resultant PPy:PSS samples were prepared as thin films of approximately 3 μm thickness, and the PPy:PSS films were cut into circular shapes with a diameter of 15 mm [6]. The PP-PE-PP trilayer membrane was cut into a circular-shaped membrane with a diameter of 17 mm [6]. The PPy:PSS electrodes and the PP-PE-PP membrane were immersed in 1 M KOH solution for 3 hours [6]. The PPy:PSS electrodes and the PP-PE-PP membrane were then assembled into coin cells and the coin cells were sealed using a hydraulic crimping machine (MSK-110, MTI Corporation, Richmond, CA, USA).

### 2.4. Electrochemical Measurements of Coin Cells Containing PPy:PSS

The electrochemical characteristics of the coin cells containing the PPy:PSS were evaluated using a ZIVE SP2 electrochemical workstation (Wonatech, Seoul, Korea). CV curves of the coin cells were measured from 0 to 1.0 V at scan rates of 1 to 50 mV/s. GCD experiments were carried out by cycling the voltages from 0 to 1.0 V at current densities from 0.05 to 2.00 A/g. The mass-specific capacitances (*C*_m_’s) of the coin cells were calculated using the equation *C*_m_ (F/g) = *I*Δ*t*/*m*Δ*V* [6,23,25]. The areal-specific capacitances (*C*_A_’s) of the coin cells were calculated using the equation *C*_A_ (mF/cm^2^) = *I*Δ*t*/*A*Δ*V* [6,23,25]. The volumetric-specific capacitances (*C*_V_’s) of the coin cells were calculated using the equation *C*_V_ (F/cm^3^) = *I*Δ*t*/*L*Δ*V* [6,23,25]. In the equations of the *C*_m_, *C*_A_, and *C*_V_, the terms *I*, Δ*t*, Δ*V*, *A*, and *L* refer to the current, discharge time, voltage, electrode area, and electrode volume, respectively. The calculation of the energy density (*E*) of the coin cells was conducted according to the equation *E* (Wh/kg) = *C*_m_Δ*V*^2^/2, where *C*_m_ and Δ*V* refer to the mass capacitance of the coin cell and the voltage drop upon discharge, respectively [6,23]. The calculation of the power density (*P*) of the coin cell was conducted using the equation *P* (W/cm^3^) = *E*/*t*, where *E* and *t* refer to the energy density and discharge time, respectively [6,23]. EIS of the coin cells was monitored in the frequency range of 1 to 10 MHz.

## 3. Results

**Figure 1:**
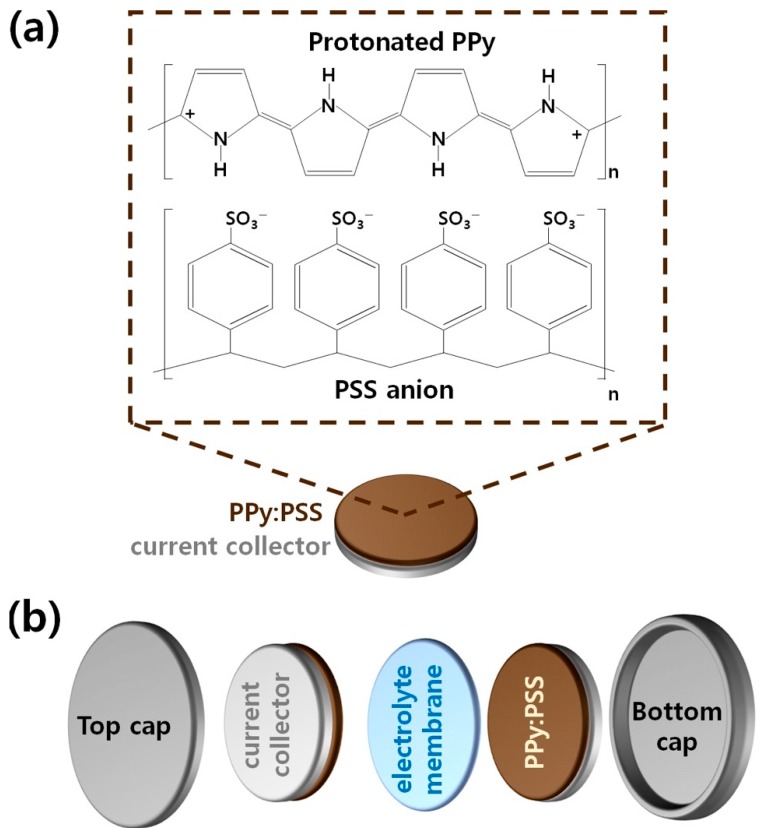
Schematic illustrations of (**a**) A Polypyrrole:Poly(4-styrenesulfonate) (PPy:PSS) electrode, and (**b**) The structure of a two-electrode symmetric supercapacitor based on a coin cell.

Figure 1a shows the molecular structure of the PPy:PSS electrode used in this research. PPy:PSS was prepared by chemical oxidative polymerization of pyrrole monomers using FeCl_3_·6H_2_O, and the PPy chain contains two cations per repeating unit. This implies that the conductive bands inside the PPy molecule are formed by overlapping bipolarons. Additional enhancement of PPy:PSS conductivity was achieved by adjusting the polymerization temperature to 3 °C. According to previous reports, the *para*-directed polymerization of conducting polymers is promoted by a lower polymerization temperature [13]. Therefore, the *para*-directed polymerization of pyrrole results in more thermodynamically stable head-to-tail structures, thereby effectively suppressing the undesirable α,α’-linkages inside PPy:PSS. PSS can be simply prepared by free radical polymerization in a water solvent [11,12,15,19,28,29]. PSS promotes the head-to-tail formation of the PPy chains and improves the dispersibility of PPy in the solvent [11,12,15,19,28,29,31,32,33]. In addition, the sulfonate anions of PSS can graft electrostatically to the cationized PPy chain, enabling graft co-polymerization between PPy and PSS [11,12,15,19,28,29,31,32,33]. Using a spin-coater, the PPy:PSS copolymers were deposited well on a nickel (Ni) substrate, in the form of thin films with a thickness of 3 μm. The overall structure of the coin cell used in this work is illustrated in Figure 1b. The coin cell consisted of two PPy:PSS electrodes, an electrolyte separator, a top cap, and a button cap, which were assembled in the form of a symmetrical supercapacitor. Ni foils were used as current collectors to facilitate PPy:PSS current generation. A polypropylene-polyethylene-polypropylene (PP-PE-PP) trilayer membrane was placed between the PSS electrodes to provide a porous path for the proper electrolyte ion transport. The PP-PE-PP membrane was immersed in a 1 M KOH aqueous solution electrolyte.

**Figure 2:**
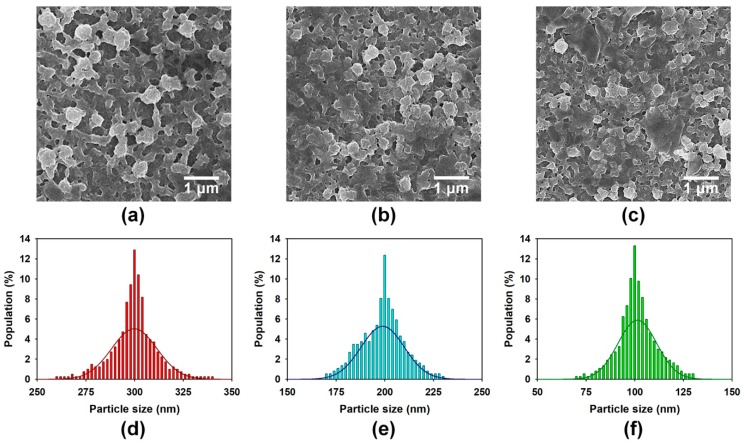
Field Emission Scanning Electron Microscope (FE-SEM) images of Polypyrrole:Poly(4-styrenesulfonate) (PPy:PSS) with different average molecular weights of PSS: (**a**) PPy:PSS with a PSS molecular weight of 1.0 × 10^6^ g/mol (PPy100), (**b**) PPy:PSS with a PSS molecular weight of 2.0 × 10^5^ g/mol (PPy20), and (**c**) PPy:PSS with a PSS molecular weight of 7.0 × 10^4^ g/mol (PPy7). Histograms for the particle size distribution of (**d**) PPy100 (**e**) PPy20, and (**f**) PPy7. The number of PPy:PSS particles analyzed were 200 each.

Field Emission Scanning Electron Microscope (FE-SEM) images of PPy:PSS electrodes, fabricated using PSS with different molecular weights, are shown in Figure 2. A large number of pores were observed on the surfaces of every sample, and the presence of these pores promotes the adsorption and desorption of electrolyte ions [10] (Figure 2a–c). The average diameter and the diameter distribution of the PPy:PSS samples were identified by histograms of the particle size distribution (Figure 2d–f). The average particle sizes of the samples containing PSS with average molecular weights of 1.0 × 10^6^ (PPy100), 2.0 × 10^5^ (PPy20), and 7.0 × 10^4^ g/mol (PPy7) were 300 ± 40, 200 ± 30, and 100 ± 30 nm, respectively. These results indicate that the size of the PPy particles increases with an increased molecular weight of PSS. Larger sizes of PPy particles are believed to enhance inter-particle connectivity, resulting in improved electron transport in supercapacitor devices [11,12,15,19,28,29,31,32,33]. Taking these facts into consideration, the PPy100 with the highest PSS molecular weight was expected to exhibit the best electrical and electrochemical properties.

**Figure 3:**
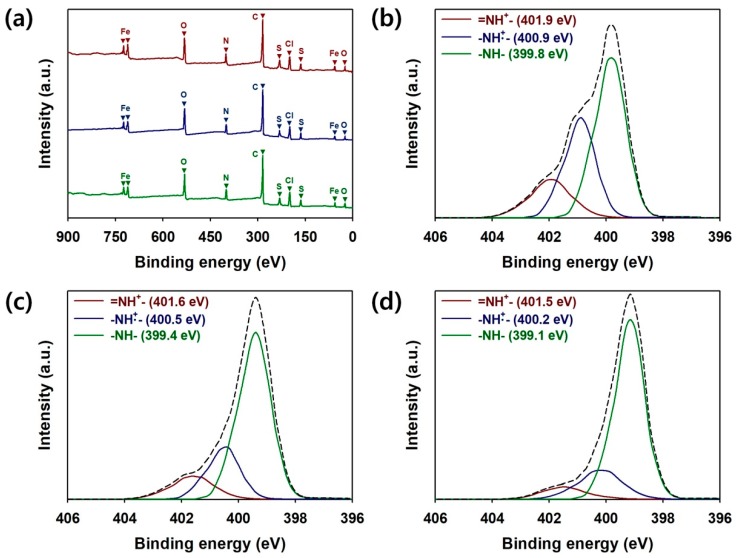
(**a**) The fully scanned X-ray photoelectron spectroscopy (XPS) spectra of PPy:PSS with a PSS molecular weight of 1.0 × 10^6^ g/mol (PPy100 (red)), PPy:PSS with a PSS molecular weight of 2.0 × 10^5^ g/mol (PPy20 (blue)), and PPy:PSS with a PSS molecular weight of 7.0 × 10^4^ g/mol (PPy7 (green)). The XPS core spectra in the N(1s) region of (**b**) PPy100, (**c**) PPy20, and (**d**) PPy7.

XPS was used to observe the changes in the elemental compositions and protonation states of PPy:PSS. (Figure 3). The fully scanned XPS patterns of the PPy:PSS samples are shown in Figure 3a. Distinctive peaks corresponding to C(1s), N(1s), O(1s), O(2s), S(2p), S(2s), Fe(2p), Fe(3p), and Cl(2p) were found in the spectra of all of the samples at the binding energies of 284, 399, 531, 24, 164, 231, 711, 55, and 197 eV, respectively [6,35]. The carbon content is the highest in the elemental compositions of the samples because PPy and PSS are hydrocarbon-based polymers (Table S1, see Supplementary Materials). The peaks for N(1s) were attributed to PPy, while the S(2p) and S(2s) peaks originated from PSS [6,35]. In addition, the relatively high content of oxygen in PPy:PSS is due to the presence of the SO_3_^−^ ions of PSS and the FeCl_3_∙6H_2_O dopant [6,35]. Furthermore, the presence of FeCl3∙6H_2_O was proven by the presence of Fe(2p), Fe(3p), and Cl(2p) peaks in all of the XPS spectra. These results reconfirm that PPy:PSS with different average molecular weights can be successfully manufactured. Figure 3b–d show the N(1s) core spectra of PPy100, PPy20, and PPy7. PPy:PSS exhibited three peaks for neutral amine nitrogen (−NH−), polaron (−NH^+^), and bipolaron (=NH^+^) at 399.1−399.8, 400.2−400.9, and 401.5−401.9 eV, respectively [6,11,35]. The ratios of N^+^ species (sum of −NH^+^ and =NH^+^) to N species (sum of −NH−, −NH^+^, and =NH^+^) were calculated to estimate the protonation states of PPy:PSS [6,11,35]. These ratios, for PPy100, PPy20, and PPy7, were 0.46, 0.31, and 0.19, respectively (Table S2, see Supplementary Materials). The N^+^/N ratio of PPy100 was close to 50%, suggesting that the half-oxidized PPy was obtained in the presence of PSS with the molecular weight of 1.0 × 10^6^ g/mol [6,11,35]. These results can be explained as follows: (1) The larger PPy particles are more densely packed, improving the connectivity between the conductive regions in PPy:PSS [11,12,15,19,28,29,31,32,33]; (2) Due to the increased inter-particle connectivity, more electrons can be delocalized within PPy:PSS structures [11,12,15,19,28,29,31,32,33]; (3) The PSS with a higher molecular weight further suppresses the undesirable α,α’-linkages that disrupt the delocalization of π-electrons inside the PPy chains [1,3,4,5,6,21]; (4) Accordingly, the effective delocalization of π-electrons in the PPy structure is due to the increased molecular weight of PSS and enables the improved protonation level of PPy:PSS.

**Figure 4:**
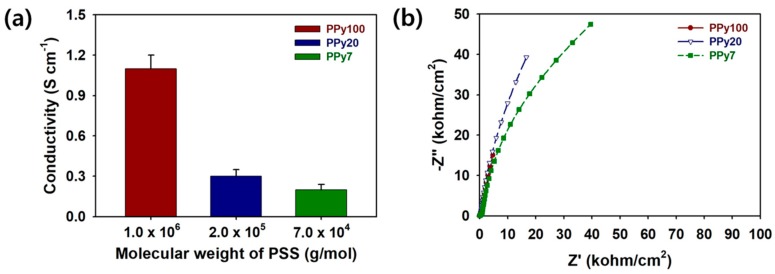
(**a**) The electrical conductivity of Polypyrrole:Poly(4-styrenesulfonate) (PPy:PSS) with different average molecular weights of 1.0 × 10^6^ g/mol (PPy100), 2.0 × 10^5^ g/mol (PPy20), and 7.0 × 10^4^ g/mol (PPy7). (**b**) Nyquist impedance plots of the coin cells containing PPy:PSS with different average molecular weights of PPy100, PPy20, and PPy7 in the frequency range from 1 to 10 MHz.

An understanding of the electrical properties of the PPy100, PPy20, and PPy7 was obtained by carrying out four-point probe measurements and EIS analyses. The results of the conductivity measurements for PPy100, PPy20, and PPy7 are presented in Figure 4a. The PPy:PSS conductivity was evaluated using the four-point probe method, as described by σ (S/cm) = 1/*ρ* = (ln2)/(π*t*)1/*R*, where *ρ*, *R*, and *t* are the static resistivity, sheet resistivity, and thickness of the thin films, respectively [34]. The PPy:PSS conductivities were 1.10 ± 0.10, 0.30 ± 0.05, and 0.20 ± 0.04 S/cm for the PSS molecular weights of 1.0 × 10^6^, 2.0 × 10^5^, and 7.0 × 10^4^ g/mol, respectively. PPy:PSS with a higher PSS molecular weight offers enhanced charge transport properties due to the higher doping levels of the PPy structure, as shown in Figure 3b [6,11,12,15,19,28,29,35]. To further identify the effects of PPy:PSS on the electrical properties of the supercapacitors, Nyquist plots of the assembled coin cells containing PPy:PSS were measured using EIS in the frequency range from 1 to 10 MHz (Figure 4b). PPy100 exhibits a much smaller semi-circle compared to PPy20 and PPy7 [6,23,24]. This implies that PPy:PSS with a higher PSS molecular weight synergistically combines the effects of high inter-particle connectivity and improved electrical conductivity, enabling more efficient charge transport on the PPy:PSS surface [6,23,24]. Therefore, among the different samples, the coin cell using PPy100 showed the smallest charge transfer resistance (*R_ct_*). In the low-frequency region, vertical straight lines were found in every Nyquist plot. The vertical straight lines are indicative of the effective ion diffusion and proper capacitive behaviors of the PPy:PSS electrodes [6,23,24]. The equivalent series resistance (ESR) of the coin cells with different PSS molecular weights increased in the order of PPy100 (2.61 Ω/cm^2^) < PPy20 (6.47 Ω/cm^2^) < PPy7 (49.5 Ω/cm^2^). It was evident that the impedances were prevented by increasing the PSS molecular weight, thereby lowering the resistance to electrolyte ions. Considering these results, PPy100 was advantageous for achieving high electrical properties superior to those of PPy20 and PPy7.

**Figure 5:**
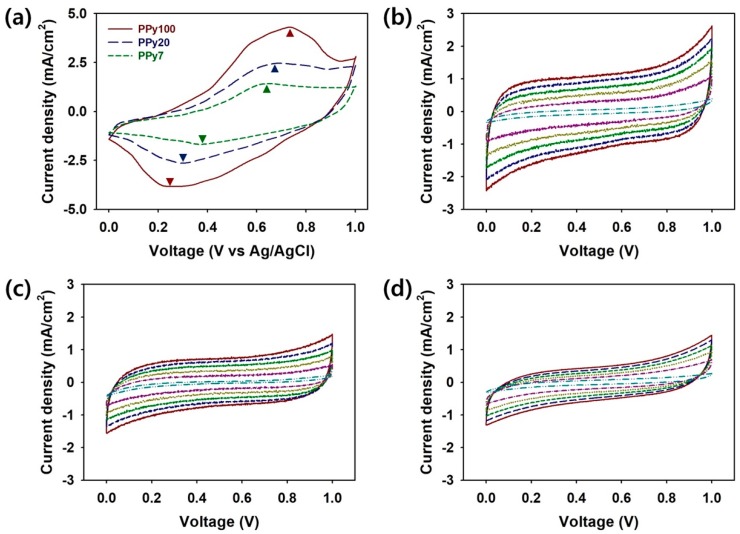
(**a**) Cyclic voltammetry (CV) curves of Polypyrrole:Poly(4-styrenesulfonate (PPy:PSS) with a PSS molecular weight of 1.0 × 10^6^ g/mol (PPy100 (red)), PPy:PSS with a PSS molecular weight of 2.0 × 10^5^ g/mol (PPy20 (blue)), and PPy:PSS with a PSS molecular weight of 7.0 × 10^4^ g/mol (PPy7 (green)) at a scan rate of 20 mV/s in 1 M KOH. CV curves of coin cells employing (**b**) PPy100, (**c**) PPy20, and (**d**) PPy7 at different scan rates: 50 mV/s (red), 40 mV/s (blue), 30 mV/s (green), 20 mV/s (olive green), 10 mV/s (purple), and 1 mV/s (cyan).

**Figure 6:**
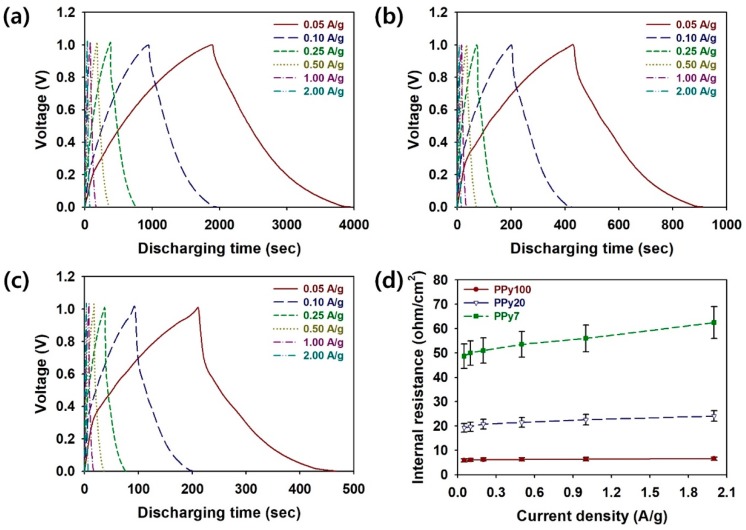
Galvanostatic charge/discharge (GCD) curves of coin cells employing (**a**) Polypyrrole:Poly(4-styrenesulfonate (PPy:PSS) with a PSS molecular weight of 1.0 × 10^6^ g/mol (PPy100), (**b**) PPy:PSS with a PSS molecular weight of 2.0 × 10^5^ g/mol (PPy20), and (**c**) PPy:PSS with a PSS molecular weight of 7.0 × 10^4^ g/mol (PPy7) at current densities. (**d**) Internal resistance values of coin cells employing Polypyrrole:Poly(4-styrenesulfonate) (PPy:PSS) with different average molecular weights at different current densities.

**Figure 7:**
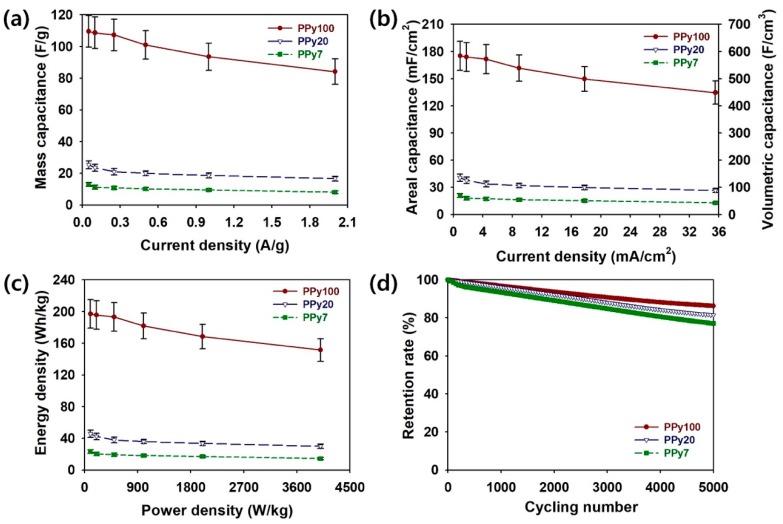
(**a**) Plots of the mass capacitance (F/g) of coin cells containing Polypyrrole:Poly(4-styrenesulfonate (PPy:PSS) with different average molecular weights at different current densities. (**b**) Plots of the areal capacitance (mF/cm^2^) and volumetric capacitance (F/cm^3^) of coin cells containing PPy:PSS with different average molecular weights at different current densities. (**c**) Ragone plots of the energy density versus the power density of coin cells containing PPy:PSS with different average molecular weights. (**d**) The cycling stability of coin cells containing PPy:PSS with different average molecular weights.

The electrochemical performance characteristics of the PPy:PSS coin cells were evaluated by CV, GCD, and cycling stability tests (Figure 5, Figure 6 and Figure 7). Cyclic voltammograms of PPy100, PPy20, and PPy7 samples were measured in 1 M KOH from 0 to 1.0 V at a scan rate of 20 mV/s (Figure 5a). Two distinctive PPy peaks were observed at 0.65−0.74 and 0.25−0.39 V, and these peaks were attributed to the oxidation and reduction reactions, respectively [24,27]. The anodic and cathodic peaks of the PPy:PSS electrodes may be the result of Faradaic reactions at the PPy:PSS/electrolyte surface. The area under the CV peaks obtained using PPy100 was larger than those for the PPy20 and PPy7 electrodes (Figure 5a). The anodic peaks shifted to higher voltages with increasing PSS molecular weight, while the cathodic peaks shifted to lower voltages with increasing PSS molecular weight. This suggests that redox reactions of PPy:PSS with a higher PSS molecular weight were enhanced by the increased interconnectivity between the PPy particles, resulting in larger currents at the electrodes [11,28]. CV testing of the coin cells was performed in a 1 M KOH electrolyte at scan rates of 1-50 mV/s (Figure 5b–d). The coin cell supercapacitors showed rectangular CV profiles, indicating excellent capacitive behaviors and fast response times for the PPy:PSS electrodes. Since the charging current is proportional to the scan rate, the current increased with scan rates in every CV profile [24]. Among the coin cells prepared using PSS with different molecular weights, the coin cell using PPy100 showed a larger CV area than those of PPy20 and PPy7, as shown in Figure 5a. As the PPy:PSS particle size increases, the interconnectivity between the PPy:PSS particles is enhanced [11,28]. Thus, the PPy:PSS with a higher PSS molecular weight allowed greater current flow in the coin cells. Moreover, the highly porous morphology of PPy:PSS may offer a larger surface area for interacting with electrolyte ions in the cells, as evidenced in Figure 2. Thus, the CV results proved the hypothesis that PPy:PSS with a higher PSS molecular weight will store more charge.

To further understand the effect of PPy:PSS on the capacitive performance of coin cell supercapacitors, GCD testing of the coin cells was performed at the current densities of 0.05, 0.10, 0.25, 0.50, 1.00, and 2.00 A/g for a voltage range from 0 to 1.0 V (Figure 6a–c). In the GCD curves of the coin cells, the charge and discharge curves were symmetric, and such symmetrical shapes of the charge/discharge curves are indicative of the stabilized charge and discharge currents [6,24,25]. Compared to PPy20 and PPy7, PPy100 exhibited longer discharge times for all current values. This suggests that PPy100, that has a larger particle size, provides better electrical properties and higher charge storage characteristics compared to PPy20 and PPy7 [11,12,15,19,28,29]. According to the Butler-Volmer equation, side reactions increase with current densities. Due to the increased side reactions at higher current densities, the discharge time of the coin cell decreased with increasing current density [24]. In particular, the coin cells using PPy100 exhibited longer discharge times than the cells using PPy20 and PPy7. The extended discharge time is directly related to the larger specific capacity and higher energy density of the coin cells using PPy100 (Figure 6a). Additionally, internal resistance (IR) drops could be estimated from the discharge curves of the assembled coin cells containing PPy100, PPy20, and PPy7 (Figure 6d). The IR drop of the coin cell containing PPy100 was significantly smaller than those of PPy20 and PPy7 at all current densities (Table S3, see Supplementary Materials). In addition, the IR of the cell using PPy100 increased more gradually compared to those of PPy20 and PPy7. This indicates that, due to its higher protonation level, PPy100 enables the highest conductivity among the samples, and the IR values observed in the GCD curves were consistent with the results of XPS, four-point probe, and EIS measurements (Figure 3 and Figure 4) [6,11,12,15,19,28,29,35]. The reduced voltage drops and IRs are strongly associated with the enhanced conductivity of the PPy:PSS electrodes and greatly enhance the charge storage characteristics of coin cell supercapacitors [6,11,12,15,19,28,29,35]. Therefore, it is reasonable to conclude that a coin cell supercapacitor using PPy100 is more suitable for practical use than those using PPy20 and PPy7.

Based on the GCD analyses, the specific capacity per mass (*C*_m_, F/g), area (*C*_A_, mF/cm^2^), and volume (*C*_V_, F/cm^3^) could be estimated in Figure 7a–c. The electrolyte ion diffusion inside PPy:PSS becomes more difficult with increasing scan speeds, resulting in decreases in the specific capacitances of coin cells. The maximum mass-specific capacitance (*C*_m_ (F/g)) of the PPy100 sample was about 109.5 F/g, which was superior to both the PPy20 (25.3 F/g) and PPy7 (13.0 F/g) (Figure 7a and Table S4, see Supplementary Materials). The same tendencies were observed for the areal-specific capacitance (*C*_A_) and the volumetric-specific capacitance (*C*_V_) (Figure 7b). The areal-specific capacitance (mF/cm^2^) values at a current density of 0.9 A/cm^2^ increased in the following order: PPy7 (20.8 mF/cm^2^) < PPy20 (40.5 mF/cm^2^) < PPy100 (175.3 mF/cm^2^). The volumetric-specific capacitance (F/cm^3^) values at a current density of 0.9 A/cm^2^ for PPy100, PPy20, and PPy7 were 584.2 F/cm^3^, 135.1 F/cm^3^, and 69.3 F/cm^3^, respectively. The capacitance value obtained from PPy100 decreased more slowly as the current density increased than that of PPy20 and PPy7 (Tables S5 and S6, see Supplementary Materials). This suggests that the PPy100, which has higher protonation level and better electrical conductivity, is more suitable for allowing the adsorption/desorption of electrolyte ions at higher currents [6,11,12,15,19,28,29,35]. In particular, the larger the areal and volumetric-specific capacitance values, the higher the possibility of the miniaturization of supercapacitors is in the state-of-art electronic applications. Judging from these facts, it was obvious that the PPy100 with a higher average molecular weight greatly improved the rate capability of the coin cell supercapacitor. To achieve further understanding of the practical applicability of the supercapacitors, Ragone plots of the coin cells containing PPy100, PPy20, and PPy7 are shown in Figure 7c [26]. At a power density of 100 W/kg, the energy densities of the coin cells assembled with PPy100, PPy20, and PPy7 were 197.2, 45.6, and 23.4 Wh/kg, respectively. These energy densities of PPy100, PPy20, and PPy7 samples were reduced to 151.5, 29.9, and 14.4 Wh/kg, respectively, at a power density of 4000 W/kg. This confirms that as the power density increases, the PPy100 sample exhibits slower decreases in energy density compared to the PPy20 and PPy7 samples (Table S7, see Supplementary Materials). Compared to PPy20 and PPy7, PPy100 not only stores more energy but also has excellent structural stability that enables the adsorption/desorption of electrolyte ions [6,24]. To ensure the reliability of the supercapacitors, the cycling stabilities of the coin cells containing PPy100, PPy20, and PPy7 were measured at a current density of 1.0 A/g for 5000 cycles (Figure 7d) [26]. The retention rates of the PPy100, PPy20, and PPy7 samples were reduced to 86.3%, 81.4%, and 77.0%, respectively. While capacitance losses are inevitable, the volumetric changes and chain scissions of the PPy:PSS during the charge/discharge process can be minimized by increasing the average molecular weight of PSS [1,2,3,4,5,6,7,8,9,10,11,21]. In addition, it is assumed that the use of the PP-PE-PP trilayer membrane effectively suppresses the evaporation of electrolyte ions. Accordingly, the coin cell configuration was effective for synergistically combining the advantages from each of the elements [6].

## 4. Conclusions

The present work described the comparative studies on PPy:PSS, containing PSS with different molecular weights, for use as symmetric supercapacitors based on the coin cell. PPy:PSS was readily prepared by chemical oxidation polymerization of pyrrole monomers in the presence of PSS. The size of PPy particles was adjusted by controlling the molecular weight of PSS from 7.0 × 10^4^ to 1.0 × 10^6^ g/mol. PSS not only acted as a steric stabilizer for controlling the PPy:PSS morphologies, but also greatly affected the resulting electrical and electrochemical characteristics of PPy. The data acquired from the four-point probe, XPS, CV, EIS, and GCD measurements demonstrated that the charge transport and electrochemical properties were significantly enhanced by increasing the molecular weight of the PPy:PSS electrodes. The coin cell supercapacitor containing PPy100 exhibited a mass capacitance (*C*_m_), areal capacitance (*C*_A_), and volumetric capacitance (*C*_V_) of 109.5 F/g, 175.3 mF/cm^2^, and 584.2 F/cm^3^, respectively, as well as an energy density of 197.2 Wh/Kg at a power density of 100 W/kg. In addition, the PPy100-based coin cell demonstrated improved cycling (86.3% after 5000 cycles at a current density of 1 A/g) and rate capability. These improvements were attributed to the higher doping level and better structural stability of PPy100 compared to the PPy20 and PPy7 samples. The method described herein is an attractive approach for obtaining miniaturized supercapacitors that require both high areal and high volumetric capacitances.

## Supplementary Materials

The following are available online at http://www.mdpi.com/2073-4360/11/2/232/s1, Table S1. The elemental composition of PPy:PSS with different average molecular weights, Table S2. Peak analyses of XPS core spectra in the N(1s) region of PPy:PSS with different average molecular weights, Table S3. IR values of coin cells containing PPy:PSS with different average molecular weights at different current densities, Table S4. The mass capacitance (F/g) of coin cells containing PPy:PSS with different average molecular weights at different current densities, Table S5. The areal capacitance (mF/cm^2^) of coin cells containing PPy:PSS with different average molecular weights at different current densities, Table S6. The volumetric capacitance (F/cm^3^) of coin cells containing PPy:PSS with different average molecular weights at different current densities, Table S7. The energy density (*E*, Wh/kg) of coin cells containing PPy:PSS with different average molecular weights at different power densities (*P*, W/kg).
