# Supplementary material for: Comparative Studies on Two-Electrode Symmetric Supercapacitors Based on Polypyrrole:Poly(4-styrenesulfonate) with Different Molecular Weights of Poly(4-styrenesulfonate)
Source: Polymers (Basel). 2019 Feb 1;11(2):232. doi: 10.3390/polym11020232 (PMC6419067; doi:10.3390/polym11020232)
Supplement: Supplementary file 1 [file polymers-11-00232-s001.pdf]

Supplementary Materials for:

Comparative Studies on Two-Electrode Symmetric Supercapacitor based on Polypyrrole:Poly(4-styrenesulfonate) with different molecular weight of Poly(4-styrenesulfonate)

Hoseong Han[1], Jun Seop Lee[2] and Sunghun Cho[1,*]

[1] School of Chemical Engineering, Yeungnam University, Gyeongsan 38541, Republic of Korea.

[2] Department of Materials Science and Engineering, College of Engineering, Gachon University, Seongnam 13120, Republic of Korea.

*E-mail: shcho83@ynu.ac.kr
Tel.: +82-53-810-2535

Table S1. Elemental composition of PPy:PSS with different average molecular weights.

Sample	Atomic ratio (%)					
	C	N	O	S	Cl	Fe
PPy100	54.30	8.11	21.17	6.95	4.87	4.60
PPy20	55.91	6.93	21.54	7.19	4.74	3.69
PPy7	56.12	8.17	21.38	5.90	5.19	3.24

Table S2. Peak analyses of XPS core spectra in the N(1s) region of PPy:PSS with different average molecular weights.

Sample	Peak ratio[a]			
	–NH–	$–NH^{\bullet +}$	$=NH^{+}$	N^{+}/N ratio[1]
PPy100	0.54	0.33	0.13	0.46
PPy20	0.69	0.21	0.10	0.31
PPy7	0.81	0.13	0.06	0.19

[a]Values were calculated using the N(1s) core spectra of the samples.

Table S3. IR values of coin cells employing PPy:PSS with different average molecular weights at different current densities.

Sample	Internal resistance (IR, ohm/cm^2)[a]					
	0.05 A/g	0.10 A/g	0.25 A/g	0.50 A/g	1.0 A/g	2.0 A/g
PPy100	5.88 ± 0.60	6.02 ± 0.60	6.21 ± 0.65	6.29 ± 0.65	6.37 ± 0.70	6.58 ± 0.70
PPy20	19.2 ± 1.8	19.7 ± 1.9	20.7 ± 2.0	21.5 ± 2.0	22.6 ± 2.2	24.1 ± 2.2
PPy7	48.7 ± 5.0	50.0 ± 5.0	51.0 ± 5.2	53.6 ± 5.3	56.0 ± 5.5	62.5 ± 6.6

[a]Values were calculated from the IR drops observed in the GCD curves.

Table S4. Mass capacitance (F/g) of coin cells employing PPy:PSS with different average molecular weights at different current densities.

Sample	Mass capacitance (C_m, F/g)[a]					
	0.05 A/g	0.10 A/g	0.25 A/g	0.50 A/g	1.0 A/g	2.0 A/g
PPy100	109.5 ± 10.0	108.7 ± 9.8	107.3 ± 9.6	101.0 ± 9.0	93.5 ± 8.5	84.2 ± 8.0
PPy20	25.3 ± 2.5	23.5 ± 2.2	21.0 ± 2.0	20.0 ± 1.9	18.6 ± 1.8	16.6 ± 1.5
PPy7	13.0 ± 1.3	11.2 ± 1.2	10.8 ± 1.0	10.1 ± 1.0	9.42 ± 0.90	8.01 ± 0.80

[a]Values were calculated from the IR drops observed in the GCD curves.

Table S5. Areal capacitance (F/cm²) of coin cells employing PPy:PSS with different average molecular weights at different current densities.

Sample	Areal capacitance (C_A, F/cm²)[a]					
	0.9 A/cm²	1.8 A/cm²	4.5 A/cm²	9.0 A/cm²	18.0 A/cm²	36.0 A/cm²
PPy100	175.3 ± 16.0	173.9 ± 15.7	171.6 ± 15.4	161.6 ± 14.4	149.7 ± 13.6	134.6 ± 12.8
PPy20	40.5 ± 4.0	37.7 ± 3.5	33.6 ± 3.2	31.2 ± 3.0	29.8 ± 2.8	26.6 ± 2.4
PPy7	20.8 ± 2.1	18.0 ± 1.9	17.2 ± 1.8	16.2 ± 1.6	15.1 ± 1.4	12.8 ± 1.3

[a]Values were calculated from the IR drops observed in the GCD curves.

Table S6. Volumetric capacitance (F/cm³) of coin cells employing PPy:PSS with different average molecular weights at different current densities.

Sample	Volumetric capacitance (C_V, F/cm³)[a]					
	0.9 A/cm²	1.8 A/cm²	4.5 A/cm²	9.0 A/cm²	18.0 A/cm²	36.0 A/cm²
PPy100	584.2 ± 53.3	579.5 ± 52.9	572.1 ± 52.0	538.7 ± 48.0	498.9 ± 13.6	448.8 ± 42.7
PPy20	135.1 ± 13.3	125.5 ± 11.7	112.0 ± 10.7	106.5 ± 8.0	99.2 ± 7.7	88.6 ± 7.4
PPy7	69.3 ± 6.9	59.8 ± 6.4	57.4 ± 5.3	53.9 ±5.1	50.3 ± 4.8	42.7 ± 4.3

[a]Values were calculated from the IR drops observed in the GCD curves.

Table S7. Energy density (E, Wh/kg) of coin cells employing PPy:PSS with different average molecular weights at different power densities (P, W/kg).

Sample	Energy density (E, Wh/kg)[a]					
	100 W/kg	200 W/kg	500 W/kg	1000 W/kg	2000 W/kg	4000 W/kg
PPy100	197.2 ± 18.0	195.6 ± 17.8	193.1 ± 17.6	181.8 ± 16.2	168.4 ± 15.3	151.5 ± 14.4
PPy20	45.6 ± 4.5	42.4 ± 4.0	37.8 ± 3.6	35.9 ± 2.7	33.5 ± 2.6	29.9 ± 2.4
PPy7	23.4 ± 2.3	20.1 ± 2.2	19.4 ± 1.8	18.2 ± 1.7	17.0 ± 1.6	14.4 ± 1.4

[a]Values were calculated from the mass capacitance, voltage drop, and discharge time observed in the GCD curves.
